# Influence of dietary supplementation of autolyzed whole yeast and yeast cell wall products on broiler chickens

**DOI:** 10.5713/ajas.19.0220

**Published:** 2019-07-01

**Authors:** Emmanuel Uchenna Ahiwe, Medani Eldow Abdallh, Edwin Peter Chang’a, Apeh Akwu Omede, Mohammed Al-Qahtani, Harriet Gausi, Hadden Graham, Paul Ade Iji

**Affiliations:** 1 School of Environmental and Rural Sciences, University of New England, Armidale NSW, 2351 Australia; 2 Department of Animal Science and Technology, Federal University of Technology, Owerri, PMB 1526, Imo State, Nigeria; 3 Department of Poultry Production, University of Khartoum, Khartoum 13314, Sudan; 4 Tanzania Livestock Research Institute (TALIRI), P. O. Box 352, Mwanza, Tanzania; 5 Department of Animal Production, Kogi State University, Anyigba, PMB 1008, Kogi State, Nigeria; 6 AB Vista UK, Marlborough, Wiltshire, SN8 4AN, UK; 7 College of Agriculture, Fisheries and Forestry, Fiji National University, P.O. Box 1544, Nausori, Fiji

**Keywords:** Chickens, Yeast, Performance, Ileal Digestibility, Enzyme Activities, Meat Yield

## Abstract

**Objective:**

This study evaluated the effect of yeast products on growth performance, visceral organ weights, endogenous enzyme activities, ileal nutrient digestibility and meat yield of broiler chickens fed diets containing autolyzed whole yeast (WY) and yeast cell walls (YCW) at varying levels of inclusion.

**Methods:**

Nine dietary treatments consisting of WY or YCW included at 0.5, 1.0, 1.5, or 2.0 g/kg diet and a control diet without yeast supplementation was used in the experiment. Each of the nine treatments was replicated six times with nine birds per replicate. Birds were housed in cages, in climate-controlled rooms and fed starter, grower and finisher diets.

**Results:**

There was an improvement (p<0.05) in body weight gain and feed conversion ratio on d 10, 24, and 35 for birds fed 1.0 to 2.0 g/kg WY or YCW diet. Small intestine weight was heavier on d 10 and 24 for birds on higher levels of WY and YCW compared to the control group. On d 10 and 24, there was a significant increase (p<0.05) in tissue protein content and pancreatic enzyme activities (trypsin and chymotrypsin) of birds on 1.5 to 2.0 g/kg WY and YCW diets compared to the control group. Compared to the control group, birds on WY (2.0 g/kg diet) and YCW (at 1.5 and 2.0 g/kg diet) had better (p<0.05) protein digestibility on d 24. On d 35, there was significant improvement (p<0.05) in percentage of carcass, absolute and relative breast weight for broiler chickens fed WY and YCW mostly at 2 g/kg diet compared to birds on the control diet.

**Conclusion:**

Supplementation of diets with autolyzed WY and YCW products especially at 1.5 to 2.0 g/kg diet improved broiler chicken performance and meat yield through their positive effects on ileal protein digestibility and pancreatic enzyme activities.

## INTRODUCTION

Non-therapeutic in-feed use of antibiotics in animal production has been implicated in the development of antibiotic-resistant bacteria, some of which are zoonotic and pathogenic to humans [1]. Based on this, the European Union, has banned the routine non-therapeutic usage of antibiotics in animal feed [2]. The continued incidence of antibiotic resistance in other regions may lead to a global ban on in-feed usage of antibiotics in animal production [3]. The benefits of in-feed antibiotics in poultry production, in terms of health and to a lesser extent, growth are well known and documented [4]. Therefore, a global ban on non-therapeutic antibiotic usage in poultry production without a suitable alternative will have an adverse effect on global poultry production [5]. Based on this premise, researchers and the global poultry feed industry have intensified efforts towards the development of suitable alternatives to in-feed antibiotics. Some probiotics (live organism) or prebiotics contains polysaccharides and thus have been reported to be potential alternatives to in-feed antibiotics in poultry production [6]. An example of such alternative is yeast and its derivatives. Yeast (*Saccharomyces cerevisiae*) and yeast cell wall (YCW) have been reported to contain polysaccharides and thus may have the potentials to improve the performance and health of birds [7]. The growth-promoting effect and immunomodulatory potential of prebiotic yeast, YCW products and their cell wall contents such as β-glucan and α-mannan in recent years are gaining research interest [6].

Yeast could be fed to livestock or poultry as probiotics (live) or prebiotics (dead/autolyzed) and no residue or pathogenic bacteria resistance effects have been reported [6,7]. Yeast and its derivatives (either as probiotic or prebiotics) contains substantial amount of energy, protein, nucleotides, vitamins, minerals, polysaccharides and some unidentified growth factors. However, different yeast products have been reported to result in different outcomes in terms of animal growth response [8,9].

A number of studies have observed the effects of yeast pro ducts as prebiotics or growth enhancer in healthy chickens [10, 11]. Others have reported its positive effect in development of gut cells, suppression of pathogenic bacteria and modulation of the immune system in challenged birds [12,13]. On the contrary, some researchers have observed that prebiotic yeast did not have effect on gross response of poultry [14,15]. These inconsistencies may be as a result of differences in processing methods used in producing the yeast product, form of the yeast product, the different yeast product composition, experimental methods used or the level of yeast product inclusion in poultry diet. So rather than consider the efficacy of yeast products as an alternative to antibiotics alone, evaluation should also be based on quality, safety, efficacy and optimum level of yeast inclusion, especially when different yeast components and different levels are tested with other sources of variability (such as environment and diet) kept constant. According to Gao et al [16], many yeast-based products have been used without proper evaluation, which leads to uncertain outcomes, in terms of broiler productivity. Studies on the systematic and comparative effects of different levels of prebiotic (autolyzed) whole yeast (WY) and YCW components on broiler performance, visceral organ weight, endogenous enzyme activities, ileal nutrient digestibility and meat yield, to the best of our knowledge are few. Furthermore, some of the mechanisms behind the improvement in broiler performance when fed with autolyzed yeast products is not very clear. Therefore, the present study was conducted to determine the level of autolyzed WY and YCW products that will provide optimal growth performance, visceral organ weight, endogenous enzyme activities, ileal nutrient digestibility and meat yield of broiler chickens.

## MATERIALS AND METHODS

### Animal ethics approval

This experiment was approved by the Animal Ethics Committee of the University of New England (Approval number: AEC17-011).

### Diet, experimental design and feeding

The broiler diets used for this trial were maize/soybean-based. The basal diets are shown in Table 1. Most of the ingredients were purchased from a local supplier in Northern New South Wales, Australia. Experimental diets, were formulated to Aviagen standards for Ross 308 [17].

Nine treatments, each with 6 replicates, and 9 birds per replicate were used in the experiment. The treatments included a control (without yeast supplementation), autolyzed WY (AB Vista, Wiltshire, UK) at 0.5, 1.0, 1.5, or 2.0 g/kg, and the YCW fraction isolated from the WY by filtration (AB Vista, Wiltshire, UK) at 0.5, 1.0, 1.5, or 2.0 g/kg in a randomized experimental design. The key nutrients in the two products include: energy (WY, 20.0, YCW, 22.0 MJ/kg dry matter); protein (WY, 58.0, YCW, 23.0%), mannan (WY, 9.2%, YCW, 24.3%), and glucan (WY, 7.4, 22.0%). Feed and water were provided *ad libitum*. The birds were provided starter crumbled diets (d 0 to 10), grower pelleted diets (d 11 to 24), and finisher pelleted diets (d 25 to 35). Titanium dioxide (TiO_2_, 5 g/kg) was incorporated in the grower diets as a marker to enable assessment of ileal nutrient digestibility.

### Birds and housing

A total of 486 day-old Ross 308 broiler chicks, with an average body weight (BW) of 40.3±2.23 g, were obtained from a local commercial hatchery (Baiada Poultry Pty. Ltd., Tamworth, Australia). Chickens were reared in multi-tiered brooder cages (600×420×23 cm^3^) placed in a climate-controlled room until the end of the trial.

The temperature of the rooms was set at 33°C for the first two days with relative humidity between 49% and 60%. This temperature was then gradually reduced to 24°C at 19 days of age and this was maintained for the remaining study period. For the first two days, 24 h of light (20 lux) was provided. This was then reduced to 23 h for the next six consecutive days, followed by 20-h light (10 lux) for the remaining duration of the experiment. The trial lasted for 35 days.

### Growth performance

On d 10, 24, and 35, the birds and feed were weighed to measure body weight gain (BWG), feed intake (FI) and feed conversion ratio (FCR; FI/weight gain). Mortality was recorded as it occurred.

### Visceral organ sampling

On d 10 and 24, one bird per cage (6 birds per treatment) was weighed, electrically stunned, killed by cervical dislocation, and key visceral organs (gizzard together with the proventriculus, heart, small intestine, pancreas, liver, spleen, and bursa) were collected, weighed and recorded. The relative weight of these organs was estimated as mass per unit of live weight (g/100 g live BW).

### Pancreatic and jejunal tissue sampling

On d10 and 24, the entire pancreas was collected from the sampled birds (one bird per cage), then snap-frozen in liquid nitrogen and stored in a freezer (−20°C) until analysis for tissue protein content and the activities of digestive enzymes. Similarly, samples (about 2 to 3 cm) from the proximal part of the jejunum, approximately 3 to 5 cm distance from the duodenum were collected, snap-frozen in liquid nitrogen and then transferred into a freezer (−20°C) and used for analysis of the tissue protein content and the activities of digestive enzymes.

### Ileal digesta sampling

Ileal digesta samples were collected at d 24, two birds were randomly selected from each cage, weighed and euthanazed using electrical stunning and cervical dislocation technique. The pooled ileal digesta content obtained from the small intestine (between the vitelline [Merkel’s] diverticulum and a point about 0.4 cm above the ileocecal junction), was flushed into a plastic container and stored at −20°C. Frozen ileal digesta samples were freeze-dried. Diet and lyophilized ileal digesta samples were then finely ground (0.5 mm pore size) using a coffee grinder and stored in air-tight containers at 4°C prior to analysis for crude protein (CP), gross energy (GE), starch, and TiO_2_ contents.

### Carcass and meat cut part sampling

At the end of the experimental period (d 35), two birds per replicate were randomly selected from each cage, weighed and euthanized by electrical stunning, followed by cervical dislocation. After de-feathering, the breast (pectoralis minor and pectoralis major), thighs and drumsticks (bones included) were separated from the carcass and weighed. The relative weights of breast, thighs and drumsticks were calculated as mass per unit live BW (g/kg of live BW).

### Gross energy, protein and starch analysis of feed and ilea digesta sample for ileal digestibility determination

The GE of the feed and ileal digesta was determined with a bomb calorimetry (IKA-Calorimeter C7000, IKA Werke GmbH & Co, Staufen, Germany) using a benzoic acid standard. The nitrogen content of ileal digesta and feed samples was determined according to the Dumas combustion technique as described by Sweeney [18], using a Leco FP-2000 analyzer and the CP content as calculated using a factor of 6.25. Starch was determined as glucose using a glucose oxidase and peroxidase method, with glyceraldehyde-3-phosphate dehydrogenase period kit supplied by Boehringer-Mannheim Australia (Castle Hill, NSW, Australia). Finely ground samples (0.5 mm) were accurately weighed into screw-capped reaction tubes (30 mL) and wet with 0.2 mL aqueous ethanol (80% v/v). A further 3 mL of thermostable α-amylase in 3-(N-morpholino) propanesulfonic acid buffer (sodium salt; Sigma M9381, Castle Hill, Australia; 50 mM, pH 7.0) were added and the samples were incubated in a boiling water bath for 6 min. After cooling, 4 mL acetate sodium buffer (200 mL, pH 4.5) were added, followed by 0.1 mL of amyloglucosidase (E.C. 3.1.1.3., Megazyme, Chicago, IL, USA) and incubated at 50°C for 30 min. Glucose was determined colorimetrically after incubating an aliquot (0.1 mL) with 3 mL of GOPOD reagent (Megazyme, USA) at 50°C for 20 min and reading the absorbance at 510 nm against a reagent blank (glucose oxidase assay).

### Ileal digestibility of nutrients determination

The concentration of titanium (Ti) in the ileal digesta and diets was determined using the method described by Short et al [19]. The values of the nutrient and the Ti marker were used to calculate the ileal digestibility as follows:

$$\text{Digestibility coefficient}=1-\frac{\text{Digesta nutrient }(\text{g}/\text{kg})/\text{digesta Ti }(\text{g}/\text{kg})}{\text{Diet nutrient }(\text{g}/\text{kg})/\text{diet Ti }(\text{g}/\text{kg})}$$

### Tissue (pancreas and jejunum) processing for the determination of enzyme activities

To evaluate the activity of digestive enzyme and protein concentration, the pancreas or jejunal tissue was processed according to the method described by Susbilla et al [20]. The supernatant from the pancreas and the homogenate from the jejunal samples were transferred into Eppendorf tubes (1.5 mL) in duplicates and stored in a freezer (−20°C) for subsequent enzyme and protein analyses.

### Determination of tissue protein content and endogenous enzyme activities

The protein content in the pancreas and jejunal mucosa was determined using the method described by Bradford [21]. The pancreatic trypsin and chymotrypsin activities were assessed using methods described by Erlanger et al [22] and modified by Caviedes-Vidal and Karasov [23]. Aminopeptidase activity was assessed as described by Caviedes-Vidal and Karasov [23]. Sucrase activity of the jejunum was determined using the method described by Iji et al [24]. Approximately 25 μL of the homogenate was incubated in a prepared solution containing 100 mg of sucrose+4 mM sodium succinate+90 mM NaCl/L of Milli-Q water, pH 6.0. The reaction was terminated with 2.5 mL of another solution containing 0.2% Triton X-100 w/v and 0.5 M Tris-HCl buffer, pH 7.02 at 37°C. Approximately 0.4 mL of this mixture was then added to 2.5 mL of GoPoD solution and was further incubated at room temperature after which absorbance was read at 610 nm. The maltase activity in the jejunum was determined using similar procedures described for sucrose, but maltose was used as substrate.

### Statistical analysis

One-way analysis of variance of Minitab software version 17 (Minitab Inc., State College, PA, USA)[25] was used to compare the mean values of treatments. The differences between the mean values were set to be significant at p≤0.05, and these mean values were also separated using Tukey’s range test. Differences between treatment groups were compared using planned orthogonal probability contrasts.

## RESULTS

### Growth performance

Throughout the study, there was no significant effect (p>0.05) of the dietary treatments on feed intake (Table 2). Compared to the control group, birds fed diets containing 2.0 g/kg WY and 1.0 to 2.0 g/kg YCW had better BWG between hatch and d 10 (Table 3). At d 24 and 35, birds in the WY and YCW groups had higher (p<0.05) BWG than the control group. Birds in the YCW group had better BWG than those in the WY group at d 35. Broilers fed diets supplemented with 1.0 to 2.0 g/kg WY or YCW recorded a higher (p<0.05) BWG compared to those fed diets without the supplements at both d 24 and 35. Birds that received diets containing higher levels (1.0 to 2.0 g/kg diet) of WY or YCW had better (p<0.05) FCR than the control group throughout the experiment (Table 4).

### Visceral organ weight

On d 10 and 24, birds in the WY and YCW group had heavier small intestine (SI) weight compared to the control group. The increase in weight of small intestine was more prominent (p< 0.05) in birds fed 2 g/kg WY or YCW diets compared to birds in the control group. (data not shown).

### Tissue protein contents and activities of digestive enzymes

Data on the effects of WY and YCW on pancreatic enzyme activities of broilers at d 10 and 24 are summarized in Table 5. Compared to birds in the control group, addition of yeast increased (p<0.05) the tissue protein content (at 1.5 and 2.0 g/kg diet), trypsin activity (at 1.0, 1.5, and 2.0 g/kg diet) and chymotrypsin activity (at 2.0 g/kg diet) at d 10. The trends observed at d 10 continued up to d 24, with the yeast supplemented groups overall having increased (p<0.05) tissue protein content (at 1.0, 1.5, and 2.0 g/kg diet), trypsin and chymotrypsin activities at 2.0 g/kg diet.

### Ileal digestibility of nutrients

Table 6 shows the data for ileal digestibility of nutients for birds fed WY or YCW compared to the control group. Birds in the WY and YCW group had higher ileal protein digestibility than birds in the control group. Birds in the YCW group had slightly (p≤0.05) higher ileal protein digestibility than those in the YCW group. Addition of 2.0 g/kg diet of WY and 1.5 or 2.0 g/kg YCW resulted in an increase (p<0.05) in the ileal protein digestibility compared to the control group.

### Carcass and meat yield

Dressing percentage was higher (p<0.05) in the WY and YCW groups fed 2.0 g/kg diet compared to the control group (Table 7). Absolute breast weight was also increased (p<0.05) with increase in WY and YCW, especially at 1.5 and 2.0 g/kg inclusion levels compared to birds in the control group. The relative breast weight (g/kg of carcass weight) was highest (p<0.05) at 2.0 g/kg inclusion levels for both WY and YCW treatment groups on d 35 (Table 8).

## DISCUSSION

### Growth performance

During the starter, grower and finisher phases, addition of both yeast products at higher levels (1.5 and 2.0 g/kg) resulted in an improvement in BWG and FCR. The improvement in BWG and FCR at a higher level of inclusion of WY or YCW compared to the control group in this study could be linked to improved gut health that led to better feed conversion as well as an associated increase in pancreatic enzyme activities (trypsin and chymotrypsin) that may have partly led to a corresponding increase in protein digestibility and nutrient utilization. Furthermore, yeast is high in nutrients and thus may serve as substrate for beneficial microbes which leads to an increase in proliferation of these gut microbes [8]. It is important to note that intestinal bacteria that exist in the gut play a vital role in digestion and absorption of feed consumed by animals, and may influence FCR [8]. These beneficial microbes may have assisted in protein digestion in the gut and led to more protein availability observed in the present study. These available nutrients when absorbed tends to help in tissue building that may reflect in broiler growth. The result on BWG and FCR in the present study show that both yeast and YCW at higher level (especially at 2 g/kg diet) can show prebiotic effect that led to improved broiler performance. The preceeding explanation could also be linked to the better BWG observed in the YCW group compared to the WY group. The concentration of polysaccharides is higher in YCW than in WY and this may have resulted in better gut health and ileal protein digestibility that culminated in better BWG. In agreement to the finding of the present study, Bradley et al [26] also observed improved lumen health, increased digestion, absorption of nutrients and growth performance, due to the effect of higher level of inclusion of yeast, *Saccharomyces cerevisiae var. boulardii* in diets fed to Turkey poults. On the contrary, Konca et al [27], noticed that finishing turkey fed diets containing 1 g/kg mannan oligosaccharide and 1 g/kg live yeast did not affect performance at 10 to 20 weeks of age. The reason behind these differences in research findings could be attributed to differences in the breed of bird used, ingredient/diet composition, form or type of yeast used as well as the age of the birds.

### Visceral organ weight

On d 24, broilers that consumed diets containing either WY (2 g/kg) or YCW (2 g/kg) had heavier SI compared to those in the control group. The WY and YCW when supplemented in broiler diet in the present study improved the ileal protein digestibility and gut health, which resulted in an improved FCR as well as an increase in BW of birds. It can therefore be postulated that the increase in SI weight could be a reflection of the increased BW of broilers fed diet containing 2 g/kg WY or YCW. The result of the present study is in agreement with the report of Santin et al [28], who observed that supplementation of 2 g/kg yeast to broiler diet enhanced nutrient digestibility, improved nutrient availability and increased villus height with a corresponding increase in the weight of the small intestine.

### Tissue protein contents and activities of digestive enzymes

Addition of autolyzed WY and YCWs at moderate (1.5 g/kg diet) and high levels (2.0 g/kg diet), increased the pancreatic tissue protein content as well as the activities of trypsin and chymotrypsin at d 10 and 24. There is substantial evidence that dietary yeast and its components may modify the morphology and structure of the intestinal mucosa, and change digestive enzyme activities and amino acid transportation in the digestive system [24]. Therefore, the improved trypsin and chymotrypsin activities may be linked to a better gut health of birds fed higher levels of WY or YCW. This may be partly associated with the observed increase in BW in the present study. This observation is in agreement with the reports of Fuller [29], who noticed that yeast can increase the digestive enzyme activities of broiler chickens. Several factors such as gut pH, microbial population and health status of the bird may probably affect the influence of yeast products on enzyme organs (i.e pancreas and jejunum). Thus these factors and their influence on enzyme activities when yeast products is fed to broilers needs further study.

### Ileal digestibility of nutrients

The observed higher ileal protein digestibility in the YCW group compared to the WY group could be due to the higher concentration of polysaccharides (present in the products) that have been reported to influence protein digestibility of birds [30]. Supplementation of 2.0 g/kg of WY and 1.5 g/kg or 2.0 g/kg of YCW in the diet resulted in an increase in the ileal protein digestibility, which may be due to the increase in pancreatic protein enzyme activities observed in the present study. The increase in ileal protein digestibility seems to be due to the higher dose administration of both WY and YCW used in the present experiment. Furthermore, one of the mode of action of yeast is its ability to influence pancreatic function, which improves the ability of the pancreas to release adequate enzymes which can assist in digestibility of dietary nutrients such as protein, and some vitamins and minerals in feed ingredients as observed in the present study. This result agrees with Chacher et al [30] who reported that dietary supplementation of broiler diet with yeast mannan oligosaccharide (MOS) product improved the protein digestibility of the birds. Likewise, addition of YCW MOS extract to poultry diets has been reported to improve protein digestibility [31].

### Carcass and meat yield

On d 35, WY and YCW supplementation at 2 g/kg diet increased the dressing percentage, while at 1.5 and 2 g/kg supplementation resulted in an increase in both absolute and relative breast weight of broiler chickens. Yeast and its components have been suggested by some authors to have the ability to decrease intestinal colonization of pathogenic bacteria by maintaining and improving intestinal health as well as mucosal integrity [24]. This action may be associated with reduced competition for nutrients between the birds and its microflora, thereby allowing more nutrients to be available to the host bird for tissue development and growth. Another possible reason to the observed increase in carcass and meat yield in the present study may be related to the role of WY and YCW at higher level of inclusion to serve as substrates for probiotic bacteria, leading to the increased proliferation of beneficial bacteria in the gut of chickens. These beneficial bacterial may aid in nutrient digestion [31] and may be partly responsible for the observed improvement in protein digestibility, availability and absorption, which resulted in the improved FCR and BW of birds as observed in the present study. Based on the preceding statement, it is note-worthy to indicate that an increase in BW of birds is associated with an increase in meat yield [32]. Thus the increased dressing % and breast meat yield at both 1.5 or 2 g/kg YW and YCW may be a reflection of the increased BWG observed in this study. This observation is in agreement with the report of Paryad and Mahmoudi [32], who reported that the inclusion of 1.5% and 2% *S. cerevisiae* significantly improved the carcass yield as well as the meat yield of broiler chickens at d 42. Contrary to the findings of the present study, Waldroup et al [33], observed that supplementation of broiler diet with MOS did not have any effect on carcass and meat yield (breast, thighs, and drumsticks) of broiler chickens. Likewise, Blair et al [34], noticed that probiotic supplementation did not affect carcass and part yields of turkey. The differences observed in carcass and meat yield between previous studies and the present study could be due to variations in the type of yeast products used, breed of birds used, the age of the birds, as well as various environmental variables.

## CONCLUSION

It can be concluded from the present study that dietary supplementation with autolyzed WY and YCW products, especially at 1.5 to 2.0 g/kg diet, improved BWG, FCR, and meat yield of broiler chickens through their positive effect on ileal protein digestibility as well as trypsin and chymotrypsin activities, even though the birds in all treatment groups consumed similar amounts of feed. Also from this study, it has been ascertained that the growth stimulating properties of autolyzed WY and YCW product are level or dosage dependent. Thus WY and YCW supplementation at 1.5 to 2 g/kg diet can exert significant prebiotic and growth promoting effect in broilers chickens.

## Figures and Tables

**Table 1:** Ingredient and nutrient composition of basal diets

Items	Feeding period (d)		

	Starter diet (0 to 10)	Grower diet (11 to 24)	Finisher diet (25 to 35)
Ingredient (g/kg)			
Corn	613.0	645.0	696.5
Soybean meal	315.0	273.5	231.3
Meat and bone meal	45.0	41.0	31.4
Canola oil	44.0	17.0	23.0
Limestone	7.7	7.1	7.1
Dicalcium phosphate	1.3	0.1	0.1
Quantum Blue 5G (100 g)	0.1	0.1	0.1
Sodium chloride	1.0	1.1	1.4
Sodium bicarbonate	1.3	1.3	1.3
Titanium dioxide	-	5.0	-
Choline Cl 70%	1.1	1.3	1.1
L-lysine HCl	3.0	2.7	2.5
DL-methionine	3.7	2.0	1.8
L-threonine	1.8	1.4	1.1
Econase GT	0.1	0. 1	0.1
UNE vitamins1)	0.6	0.6	0.6
UNE minerals2)	0.8	0.8	0.8
Nutrient composition (g/kg)			
AME (MJ/kg)3)	12.6	13.0	13.4
Crude protein	230	212	192
Crude fat	28.0	39.8	45.4
Crude fibre	20.9	24.0	23.1
Lysine	12.8	11.5	10.2
Methionine	6.60	4.70	4.30
Met+Cys	9.50	7.43	6.86
Calcium	9.60	8.70	7.80
Phosphorus available	4.80	4.40	3.90
Sodium	1.60	1.60	1.60
Potassium	9.30	8.50	7.60
Chloride	2.30	2.30	2.30
Choline	1.30	1.30	1.30

1) University of New England vitamin premix supplied per 0.6 kg/tonne of diet: retinol, 12,000 IU; cholecalciferol, 5,000 IU; tocopheryl acetate, 75 mg; menadione, 3 mg; thiamine, 3 mg; riboflavin, 8 mg; niacin, 55 mg; pantothenate, 13 mg; pyridoxine, 5 mg; folate, 2 mg; cyanocobalamin, 16 μg; biotin, 200 μg; cereal-based carrier, 149 mg.

2) Minerals premix supplied per 0.8 kg/tonne of diet: mineral oil, 2.5 mg; Cu (sulphate), 16 mg; Fe (sulphate), 40 mg; I (iodide), 1.25 mg; Se (selenate), 0.3 mg; Mn (sulphate and oxide), 120 mg; Zn (sulphate and oxide), 100 mg; cereal-based carrier, 128 mg; mineral oil, 3.75 mg.

3) AME, apparent metabolizable energy.

**Table 2:** Effects of dietary treatments on feed intake (g/bird) of chicks at different ages

Items	Inclusion levels (g/kg)	0 to 10 d	0 to 24 d	0 to 35 d
Control	0.0	336	1,652	3,231
Whole yeast	0.5	326	1,650	3,133
	1.0	319	1,604	3,143
	1.5	310	1,629	3,030
	2.0	322	1,633	3,075
Yeast cell wall	0.5	318	1,653	3,207
	1.0	324	1,637	3,197
	1.5	316	1,635	3,187
	2.0	313	1,671	3,216
SEM		3.25	8.70	19.0
p-value		0.78	0.86	0.16
Probability levels of orthogonal contrasts				
Control vs yeast		0.11	0.66	0.16
Control vs whole yeast		0.15	0.46	0.08
Control vs yeast cell wall		0.11	0.93	0.64
Whole yeast vs yeast cell wall		0.83	0.31	0.10

Values are means of 6 replicates.

SEM, standard error of the mean.

**Table 3:** Effects of dietary treatments on body weight gain (g/bird) of chicks at different ages

Items	Inclusion levels (g/kg)	0 to 10 d	0 to 24 d	0 to 35 d
Control	0.0	278^b^	1,274^c^	2,210^c^
Whole yeast	0.5	285^b^	1,275^c^	2,225^c^
	1.0	289^b^	1,302^ab^	2,317^b^
	1.5	287^b^	1,318^b^	2,318^b^
	2.0	303^a^	1,346^a^	2,368^b^
Yeast cell wall	0.5	286^b^	1,284^c^	2,294^c^
	1.0	304^a^	1,320^ab^	2,336^b^
	1.5	305^a^	1,340^a^	2,414^a^
	2.0	306^a^	1,358^a^	2,494^a^
SEM		1.94	7.10	2.10
p-value		0.01	0.01	0.01
Probability levels of orthogonal contrasts				
Control vs yeast		0.001	0.02	0.03
Control vs whole yeast		0.01	0.04	0.04
Control vs yeast cell wall		0.001	0.01	0.002
Whole yeast vs yeast cell wall		0.21	0.11	0.04

Values are means of 6 replicates.

SEM, standard error of the mean.

a–c Means in a column not sharing a common superscript differ (p<0.05).

**Table 4:** Effects of dietary treatments on feed conversion ratio (FCR, g/g) of chicks at different ages

Items	Inclusion levels (g/kg)	0 to 10 d	0 to 24 d	0 to 35 d
Control	0.0	1.18^a^	1.29^a^	1.47^a^
Whole yeast	0.5	1.14^a^	1.29^a^	1.40^a^
	1.0	1.11^a^	1.23^b^	1.36^b^
	1.5	1.08^b^	1.24^b^	1.31^b^
	2.0	1.06^b^	1.21^b^	1.30^b^
Yeast cell wall	0.5	1.12^a^	1.29^a^	1.40^a^
	1.0	1.06^b^	1.24^b^	1.37^b^
	1.5	1.04^b^	1.22^b^	1.32^b^
	2.0	1.02^b^	1.21^b^	1.29^b^
SEM		0.01	0.01	0.01
p-value		0.02	0.01	0.01
Probability levels of orthogonal contrasts				
Control vs yeast		0.006	0.01	0.001
Control vs whole yeast		0.03	0.01	0.01
Control vs yeast cell wall		0.002	0.01	0.001
Whole yeast vs yeast cell wall		0.12	0.99	0.99

Values are means of 6 replicates.

SEM, standard error of the mean.

a,b Means in a column not sharing a common superscript differ (p<0.05).

**Table 5:** Effects of dietary treatments on protein concentration (mg/g tissue) and pancreatic enzyme activity (μmol/mg protein/min) of broilers at 10 and 24 d of age

Items	Level (g/kg)	Protein		Trypsin		Chymotrypsin	
		
		10 d	24 d	10 d	24 d	10 d	24^d^
Control	0.0	36.9^b^	37.5^b^	4.1^b^	4.4^b^	4.1^b^	4.9^b^
Whole yeast	0.5	37.1^b^	39.1^b^	4.4^b^	4.6^b^	4.3^b^	4.9^b^
	1.0	42.8^ab^	41.3^b^	5.2^a^	4.5^b^	4.7^ab^	4.9^b^
	1.5	54.4^a^	51.5^a^	5.1^a^	5.1^a^	4.6^ab^	5.5^a^
	2.0	55.2^a^	54.6^a^	5.9^a^	5.4^a^	5.6^a^	5.9^a^
Yeast cell wall	0.5	40.0^ab^	41.6^b^	4.4^b^	4.1^b^	4.1^b^	4.9^b^
	1.0	45.2^a^	51.4^a^	5.4^a^	5.1^a^	4.8^ab^	5.0^b^
	1.5	49.1^a^	50.5^a^	5.5^a^	5.1^a^	4.9^ab^	5.5^a^
	2.0	54.1^a^	54.3^a^	5.6^a^	5.4^a^	5.7^a^	5.9^a^
SEM		1.48	1.45	0.15	1.45	0.14	0.09
p-value		0.01	0.004	0.02	0.004	0.04	0.002
Probability levels of orthogonal contrasts							
Control vs yeast		0.04	0.03	0.03	0.02	0.04	0.03
Control vs whole yeast		0.03	0.19	0.04	0.03	0.02	0.52
Control vs yeast cell wall		0.02	0.02	0.03	0.02	0.03	0.04
Whole yeast vs yeast cell wall		0.10	0.06	0.44	0.48	0.66	0.62

Values are means of 6 replicates.

SEM, standard error of the mean.

a,b Means in a column not sharing a common superscript differ (p<0.05).

**Table 6:** Effect of dietary treatments on nutrient ileal apparent digestibility of broiler chickens at 24 d of age

Items	Level (g/kg)	Gross energy	Protein	Starch
Control	0.0	0.60	0.64^b^	0.92
Whole yeast	0.5	0.62	0.64^b^	0.93
	1.0	0.62	0.65^b^	0.93
	1.5	0.61	0.69^ab^	0.92
	2.0	0.64	0.72^a^	0.93
Yeast cell wall	0.5	0.60	0.64^b^	0.92
	1.0	0.61	0.65^b^	0.92
	1.5	0.64	0.72^a^	0.93
	2.0	0.64	0.73^a^	0.94
SEM		0.006	0.004	0.001
p-value		0.08	0.001	0.11
Probability levels of orthogonal contrasts				
Control vs yeast		0.11	0.001	0.19
Control vs whole yeast		0.16	0.001	0.27
Control vs yeast cell wall		0.10	0.001	0.16
Whole yeast vs yeast cell wall		0.69	0.05	0.64

Values are means of 6 replicates.

SEM, standard error of the mean.

a,b Means in a column not sharing a common superscript differ(p<0.05).

**Table 7:** Effect of dietary treatments on dressing percentage and absolute meat yield (g) of broiler chickens at 35 d of age

Items	Levels (g/kg)	Dressing %	Breast	Thighs	Drumsticks
Control	0.0	73.8^b^	507.0^b^	245.0	215.6
Whole yeast	0.5	73.8^b^	518.6^b^	249.2	219.7
	1.0	75.8^ab^	545.2^ab^	258.9	221.1
	1.5	76.5^ab^	563.7^ab^	264.3	224.8
	2.0	80.7^a^	604.2^a^	267.0	216.6
Yeast cell wall	0.5	75.5^ab^	515.9^b^	249.2	230.2
	1.0	77.5^ab^	547.0^ab^	258.9	234.0
	1.5	81.8^a^	607.5^a^	271.1	234.0
	2.0	81.9^a^	637.7^a^	279.8	238.7
SEM		0.75	7.04	2.83	2.37
p-value		0.04	0.01	0.06	0.25
Probability levels of orthogonal contrasts					
Control vs yeast		0.07	0.001	0.44	0.17
Control vs whole yeast		0.04	0.001	0.10	0.44
Control vs yeast cell wall		0.02	0.001	0.28	0.07
Whole yeast vs yeast cell wall		0.06	0.08	0.35	0.10

Values are means of 6 replicates.

SEM, standard error of the mean.

a,b Means in a column not sharing a common superscript differ (p<0.05).

**Table 8:** Effect of dietary treatments on relative meat yield (g/kg) of broiler chickens at 35 d of age

Items	Levels (g/kg)	Breast	Thighs	Drumsticks
Control	0.0	212.1^b^	102.6	90.1
Whole yeast	0.5	212.1^b^	101.9	89.9
	1.0	216.6^b^	102.5	88.0
	1.5	218.9^b^	102.7	86.0
	2.0	234.0^a^	102.7	87.0
Yeast cell wall	0.5	210.5^b^	102.0	88.7
	1.0	216.6^b^	102.6	91.0
	1.5	230.0^a^	102.6	88.6
	2.0	233.9^a^	102.7	88.6
SEM		2.12	2.83	2.37
p-value		0.02	0.98	0.84
Probability levels of orthogonal contrasts				
Control vs yeast		0.04	0.95	0.99
Control vs whole yeast		0.03	0.95	0.96
Control vs yeast cell wall		0.02	0.96	0.97
Whole yeast vs yeast cell wall		0.10	0.99	0.99

Values are means of 6 replicates.

SEM, standard error of the mean.

a,b Means in a column not sharing a common superscript differ (p<0.05).
